# A role for the Cajal-body-associated SUMO isopeptidase USPL1 in snRNA transcription mediated by RNA polymerase II

**DOI:** 10.1242/jcs.141788

**Published:** 2014-03-01

**Authors:** Saskia Hutten, Georgia Chachami, Ulrike Winter, Frauke Melchior, Angus I. Lamond

**Affiliations:** 1 Centre for Gene Regulation and Expression, College of Life Sciences, University of Dundee, Dundee DD15EH, UK; 2 Zentrum für Molekulare Biologie der Universität Heidelberg (ZMBH), DKFZ-ZMBH Alliance, 69120 Heidelberg, Germany

**Keywords:** Cajal body, snRNA transcription, SUMO isopeptidase

## Abstract

Cajal bodies are nuclear structures that are involved in biogenesis of snRNPs and snoRNPs, maintenance of telomeres and processing of histone mRNA. Recently, the SUMO isopeptidase USPL1 was identified as a component of Cajal bodies that is essential for cellular growth and Cajal body integrity. However, a cellular function for USPL1 is so far unknown. Here, we use RNAi-mediated knockdown in human cells in combination with biochemical and fluorescence microscopy approaches to investigate the function of USPL1 and its link to Cajal bodies. We demonstrate that levels of snRNAs transcribed by RNA polymerase (RNAP) II are reduced upon knockdown of USPL1 and that downstream processes such as snRNP assembly and pre-mRNA splicing are compromised. Importantly, we find that USPL1 associates directly with U snRNA loci and that it interacts and colocalises with components of the Little Elongation Complex, which is involved in RNAPII-mediated snRNA transcription. Thus, our data indicate that USPL1 plays a key role in RNAPII-mediated snRNA transcription.

## INTRODUCTION

The eukaryotic cell nucleus contains multiple compartments, or bodies, including the nucleolus, PML bodies, Cajal bodies and splicing speckles (reviewed by Dundr and Misteli, 2010; Handwerger and Gall, 2006; Spector, 2001; Spector, 2006; Spector and Lamond, 2011; Zhao et al., 2009). By concentrating a defined set of protein and/or RNA components, nuclear bodies have been suggested to act as sequestration or reaction sites for specific factors and/or cellular processes, thereby regulating complex formation and gene expression (Mao et al., 2011).

Cajal bodies are dynamic structures that vary in size and number according to the cell cycle stage, differentiation and developmental status of the cell (reviewed by Cioce and Lamond, 2005; Machyna et al., 2013; Morris, 2008). They have been associated with roles in biogenesis of spliceosomal small nuclear (snRNPs) and small nucleolar RNPs (snoRNPs), maintenance of telomeres and histone mRNA processing. snRNPs represent the major constituents of the spliceosome, which is a complex machinery involved in removing introns from pre-mRNA (reviewed by Wahl et al., 2009; Will and Lührmann, 2011). Each snRNP is composed of a unique RNA component in complex with a specific set of proteins (reviewed by Fischer et al., 2011; Kiss, 2004; Patel and Bellini, 2008). Most snRNAs are transcribed by RNA polymerase (RNAP) II and require a cytoplasmic maturation step. RNAPII-transcribed snRNA gene loci have been observed in proximity to Cajal bodies (Frey and Matera, 1995; Gao et al., 1997; Jacobs et al., 1999; Smith et al., 1995), and several components involved in RNAPII transcription of snRNAs are enriched in Cajal bodies (Hu et al., 2013; Polak et al., 2003; Smith et al., 2011). Exceptions are U6 and U6atac snRNAs, which are transcribed by RNAPIII and pass through the nucleolus for their maturation process (Ganot et al., 1999; Kunkel et al., 1986; Lange and Gerbi, 2000; Reddy et al., 1987; Tycowski et al., 1998).

After export of snRNAs into the cytoplasm, the SMN complex directs the assembly of snRNAs with the common Sm proteins (B/B′, D1, D2, D3, E, F, G) (Fischer et al., 1997; Kambach et al., 1999; Massenet et al., 2002; Pellizzoni et al., 2002). Subsequently, the 5′ end of the snRNA is hypermethylated to form the TMG-cap and snRNP complexes are re-imported into the nucleus (Fischer and Lührmann, 1990; Fischer et al., 1993; Hamm et al., 1990; Mattaj, 1986; Mouaikel et al., 2003; Plessel et al., 1994). Here, they initially concentrate within Cajal bodies prior to forming the typical ‘speckled’ pattern seen for mature snRNPs (Sleeman and Lamond, 1999). In Cajal bodies, scaRNAs can mediate post-transcriptional RNA modifications and additional snRNP assembly steps can take place before snRNP complexes are released into the nucleoplasm (Darzacq et al., 2002; Jády et al., 2003; Kiss, 2001). There is evidence that the formation of di-snRNPs (U4/6 snRNP) and tri-snRNPs (U4/6/5 snRNP) as well as their reassembly after each round of splicing occurs within Cajal bodies (Schaffert et al., 2004; Stanek and Neugebauer, 2004; Stanek et al., 2008).

Coilin is generally considered a marker protein for Cajal bodies (Raška et al., 1991). Whereas knockdown of coilin has been shown to lead to defects in snRNP biogenesis and embryonic lethality in zebrafish (Strzelecka et al., 2010), neither coilin nor the presence of Cajal bodies appears to be essential for viability in mammalian cell lines, mice, *Drosophila* or *Arabidopsis* (Collier et al., 2006; Lemm et al., 2006; Liu et al., 2009; Tucker et al., 2001). However, coilin-induced Cajal bodies might serve to increase the efficiency of complex assembly by providing a structural scaffold to enrich components involved in snRNP biogenesis (Klingauf et al., 2006; Matera et al., 2009; Novotný et al., 2011).

Recently, an essential SUMO isopeptidase (USPL1) that localises to Cajal bodies has been described (Schulz et al., 2012). RNAi-mediated knockdown of USPL1 in HeLa cells leads to disruption of Cajal bodies, relocalisation of coilin to the nucleolus and impaired cell proliferation. Strikingly, these effects are not dependent on the catalytic activity of USPL1 as a SUMO isopeptidase, suggesting additional functions for USPL1 linked with the Cajal body. Therefore, we undertook a detailed study on the effects of USPL1 knockdown on nuclear function and architecture.

We show here that upon knockdown of USPL1 there are changes in the localisation and/or mobility of both snRNA- and mRNA-associated proteins and the splicing pattern of specific pre-mRNAs is altered. We demonstrate that this phenotype is associated with reduced snRNP biogenesis and low levels of RNAPII-transcribed snRNAs. We further show an interaction of endogenous USPL1 with members of the snRNA-specific transcription complex and an enrichment of USPL1 at snRNA gene loci, suggesting a key role for USPL1 in snRNA transcription.

## RESULTS

### Knockdown of USPL1 affects Cajal bodies and splicing speckles

**Fig. 1.**
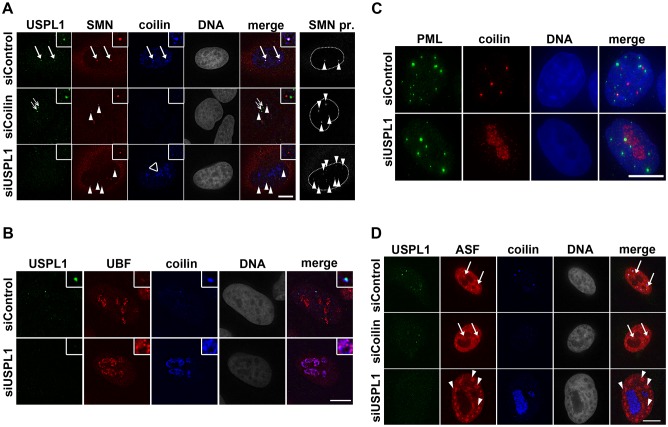
**Consequences of USPL1 knockdown on nuclear architecture.** (A) Immunofluorescence of U2OS cells transfected with siRNA as indicated were stained against USPL1, SMN and coilin. Closed arrows in top row indicate Cajal bodies with USPL1, SMN and coilin present. Open arrows in middle row highlight USPL1 nuclear foci in the absence of coilin; arrowheads (middle and bottom row) indicate SMN nuclear foci upon treatment with siRNA against coilin or USPL1. A maximum intensity projection in the right column demonstrates the increase in SMN nuclear foci (foci indicated by arrowheads, nucleus indicated by the dotted line). Similar effects were observed with HeLa cells (data not shown). (B) Immunofluorescence of siRNA transfected U2OS cells stained against USPL1, coilin and UBF. Similar effects were observed with HeLa cells. (C) A maximum intensity projection of siRNA treated HeLa cells stained with antibodies against PML and coilin. Similar effects were observed with U2OS cells (data not shown). (D) Immunofluorescence of HeLa cells transfected with siRNA as indicated were stained against ASF and coilin. Arrows highlight splicing speckles in control and coilin-depleted cells (top and middle row, respectively), arrowheads indicate enlarged splicing speckles upon USPL1 knockdown (bottom row). Similar effects were observed with U2OS cells (Fig. 7). Scale bars: 10 µm.

After siRNA-mediated knockdown of USPL1, the Cajal body marker protein coilin relocalises into the nucleolus (Schulz et al., 2012). Because other Cajal body components form nuclear bodies without coilin (Bauer and Gall, 1997; Jády et al., 2003; Lemm et al., 2006; Tucker et al., 2001), we compared the Cajal-body-associated protein SMN in control cells and in cells after knockdown of USPL1. USPL1 often colocalised in Cajal bodies with both SMN and coilin (Fig. 1A, arrows in top panel). Occasionally, we observed USPL1 in nuclear foci that did not obviously label with coilin. Upon knockdown of coilin, USPL1 still formed nuclear foci, similar to SMN (Fig. 1A, arrows in middle panel). As described previously (Schulz et al., 2012), coilin concentrates in the nucleolus after knockdown of USPL1 (Fig. 1A, open arrowhead in bottom panel). In these cells, SMN localises in a larger number of nuclear foci (Fig. 1A, arrowheads in bottom panel). USPL1 levels were efficiently reduced as detected by immunoblotting (supplementary material Fig. S1A). By contrast, levels of SMN protein appeared not or only mildly altered by siRNA against USPL1, despite the drastic changes to SMN foci in the nucleus upon knockdown of USPL1. Controls confirmed no change in the levels of coilin upon knockdown of USPL1 (Fig. 4A; supplementary material Fig. S1A) (Schulz et al., 2012).

We next tested whether other nuclear bodies might also be affected by knockdown of USPL1. Therefore, we investigated the effect of USPL1 knockdown on nucleoli, PML bodies and splicing speckles by immunostaining. Whereas in the nucleoli of some cells UBF and fibrillarin appeared less condensed upon depletion of USPL1 (Fig. 1B and supplementary material Fig. S1B, respectively), no dramatic loss of nucleolar integrity could be observed, which is consistent with earlier observations (Schulz et al., 2012). The integrity of PML bodies showed no apparent change upon knockdown of USPL1 (Fig. 1C). This suggests that knockdown of USPL1 causes only a minor effect on nucleoli and little or no effect on PML bodies.

Splicing factors are often concentrated in interchromatin granule clusters, also called nuclear speckles (reviewed by Spector and Lamond, 2011). In control cells, nuclear speckles exhibited an irregular fine-structured pattern, as shown for ASF/SF2 and Sm proteins (Y12 antibody) (arrows in top panel Fig. 1D and supplementary material Fig. S1C, respectively). By contrast, USPL1-knockdown cells displayed enlarged, rounded nuclear speckles (Fig. 1D and supplementary material Fig. S1C, arrowheads in bottom panel). In comparison, splicing speckles in cells transfected with siRNA targeting coilin did not differ from control cells, demonstrating that this effect on speckles is not caused directly by loss of Cajal bodies (Fig. 1D, arrows in middle panel).

### Knockdown of USPL1 affects splicing

**Fig. 2.**
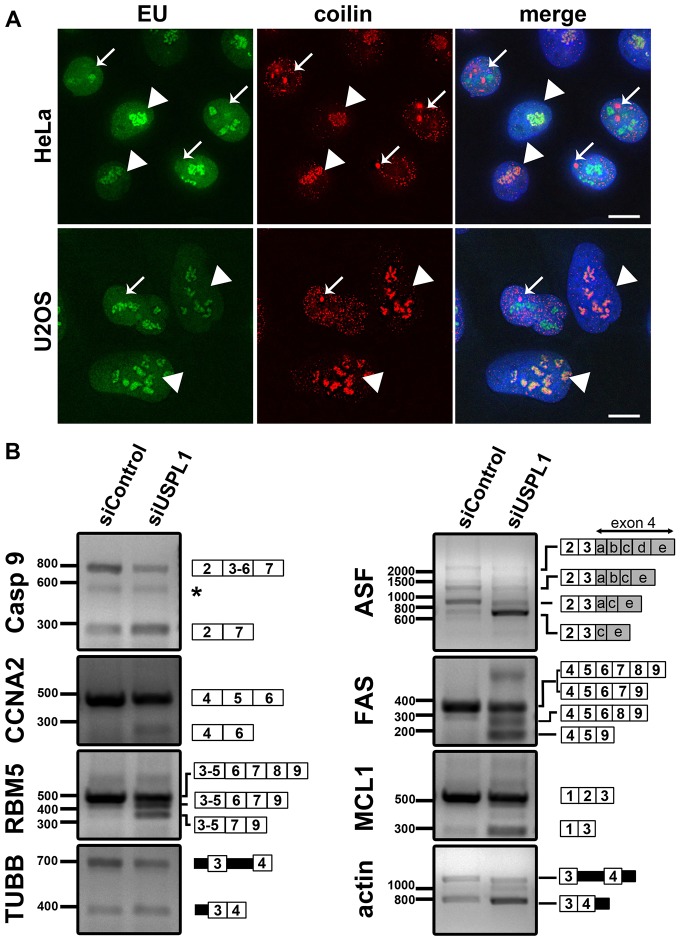
**Knockdown of USPL1 affects pre-mRNA splicing.** (A) An excess of HeLa (top panel) or U2OS (bottom panel) cells treated with siRNA against USPL1 were mixed with control cells and analysed by 5-ethynyl uridine pulse labelling to monitor nascent RNA transcription. siControl- and siUSPL1-treated cells can be distinguished by parallel immunostaining for coilin: arrows indicate control cells with coilin in Cajal bodies, whereas arrowheads highlight USPL1-knockdown cells with coilin in nucleoli. Note, to allow visibility of nucleolar coilin, Cajal-body-localised coilin was allowed to saturate the camera during image acquisition. Scale bar: 10 µm. (B) Changes in pattern of pre-mRNA splicing detected by qualitative RT-PCR on total RNA isolated from siRNA-transfected U2OS cells and subsequent ethidium bromide agarose gel electrophoresis. Similar effects were observed with HeLa cells (data not shown). Splicing products from either HeLa or U2OS USPL1-knockdown cells were sequenced and their positions are indicated next to the gel. White boxes indicate exons, filled black boxes introns, with the position of respective primers corresponding to the first and last box, respectively (size of box not to scale). An asterisk indicates a nonspecific band.

The observed reorganisation of splicing speckles into enlarged, rounded structures, typically occurs upon inhibition of transcription and/or pre-mRNA splicing (Carmo-Fonseca et al., 1992; O'Keefe et al., 1994; Spector et al., 1984). To analyse whether general transcription is affected by the knockdown of USPL1, we visualised nascent RNA synthesis by 5-ethynyl uridine labelling (Jao and Salic, 2008) (Fig. 2A). To improve comparison of transcription levels, an excess of cells transfected with the siRNA against USPL1 were mixed with cells treated with control siRNA the day before analysis (Fig. 2A). On the basis of the good correlation of nucleolar localisation for coilin with knockdown of USPL1, we used immunostaining for coilin to monitor USPL1 knockdown.

Cells with reduced levels of USPL1 (identified by nucleolar coilin stain; Fig. 2A, arrowheads) still exhibited transcriptional activity at levels either similar to, or only slightly reduced, in comparison to control cells (coilin in Cajal bodies, arrows; Fig. 2A). This is consistent with the fact that we did not observe nucleolar cap formation upon knockdown of USPL1, which would be expected upon a general inhibition of transcription (Carmo-Fonseca et al., 1992; Shav-Tal et al., 2005).

Because a general loss of transcription did not cause the enlarged nuclear speckles upon USPL1 knockdown, we next analysed pre-mRNA splicing of endogenous pre-mRNAs extracted from either control, or USPL1-knockdown cells (Fig. 2B). We detected changes in the alternative splicing pattern of several pre-mRNAs by qualitative RT-PCR and agarose gel electrophoresis (CCNA2, FAS, MCL1, RBM5, ASF/SF2 and Caspase 9 mRNA; Fig. 2B). Additionally, using a combination of primers located in introns and exons of pre-mRNA, we detected different ratios of intermediate splicing products of the constitutively spliced pre-mRNAs encoding β-tubulin and β-actin (Fig. 2B). This demonstrates that the pattern of pre-mRNA splicing was altered following USPL1 knockdown in this cell system, at least for a subset of pre-mRNAs.

### Knockdown of USPL1 alters the localisation and abundance of snRNP components

**Fig. 3.**
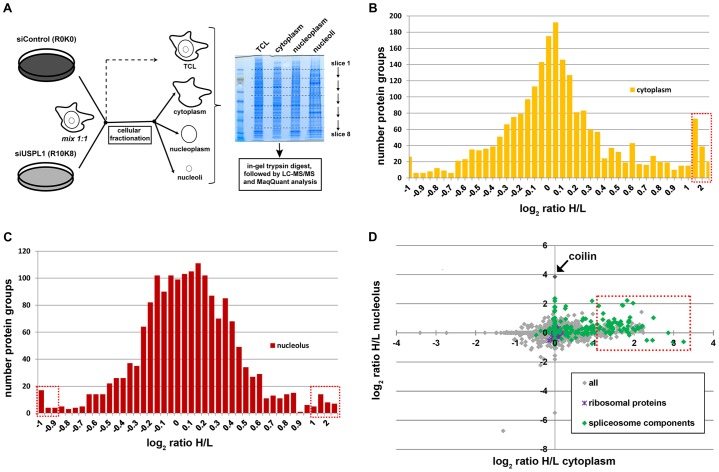
**SILAC MS-based analysis reveals impact of USPL1 knockdown on localisation and mobility of spliceosome-associated proteins.** (A) Experimental workflow of the cellular fractionation process and 1D SDS-PAGE for subsequent SILAC-MS analysis upon USPL1 knockdown. (B–D) SILAC analysis of cellular fractionation of HeLa cells upon USPL1 knockdown. Only hits identified with >1 peptide in an individual fraction are displayed here. The log_2_ ratio heavy (H; siUSPL1)/light (L; siControl) of each cellular fraction is displayed as frequency histogram for cytoplasm (B) and nucleolus (C). Protein groups above/below the arbitrary threshold are indicated by a red lined box (B,C). The log_2_ ratios [H (siUSPL1)/L (siControl)] of the cytoplasmic versus nucleolar fraction are displayed as a scatter plot with each dot representing an individual protein (D). Spliceosome proteins (green, based on Hegele et al., 2012) and ribosomal proteins (purple) are highlighted as indicated. The red lined box highlights protein groups enriched in the cytoplasmic fraction. Note, for clarity, the three major environmental contaminants (keratins) are not displayed in D.

We next analysed whether there were changes in overall protein abundance and localisation after siRNA knockdown of USPL1, combining siRNA knockdown with stable isotope labelling by amino acids in cell culture (SILAC)-based mass spectrometry (MS) (Ong et al., 2002) and cellular fractionation. After metabolic labelling, HeLa cells were transfected with the respective siRNAs (R_0_K_0_/light: siControl; R_10_K_8_/heavy: siUSPL1). Equal numbers of cells were mixed and fractionated into cytoplasm, nucleoplasm and nucleoli, as previously described (Andersen et al., 2002; Boisvert et al., 2010) (Fig. 3A). The knockdown and fractionation efficiency was checked by immunofluorescence staining for coilin and immunoblot analysis, respectively (data not shown).

The individual fractions were analysed by 1D-SDS-PAGE and the peptides obtained from in-gel tryptic digestion analysed by LC-MS/MS (see supplementary material Table S1). To relate changes of protein levels in subcellular fractions to possible changes in overall protein levels, total cell lysate (TCL) was analysed in parallel. When displayed as a histogram of the log_2_ ratio H/L (Fig. 3B,C and supplementary material Fig. S2A,B), most protein groups from the cellular fractions fell into a distribution centred around zero, indicating that their intracellular localisation is unaffected by knockdown of USPL1. However, ∼5% of all proteins identified in the cytoplasm and ∼2% of all proteins in the nucleolar fraction were more abundant in these fractions upon knockdown of USPL1 (indicated by red box in Fig. 3B,C). As expected, we found that coilin was enriched in the nucleolar fraction (log_2_ H/L ratio nucleolus ∼4, cytoplasm N/A; Fig. 3D), confirming our previous immunofluorescence data showing its nucleolar relocalisation upon knockdown of USPL1.

An enrichment analysis based on functional annotation for proteins using the bioinformatics database DAVID (Huang et al., 2009a; Huang et al., 2009b) revealed a highly significant enrichment for proteins associated with the spliceosome and RNA splicing in the cytoplasmic and/or nucleolar fractions upon knockdown of USPL1 [corrected *P*-value (Benjamini): <1×10^−10^; supplementary material Table S2]. This enrichment is illustrated by highlighting splicing factors in Fig. 3D in green (see list of protein group IDs in supplementary material Table S1).

When analysed by immunostaining, none of the non-snRNP proteins displaying enrichment in the cytoplasm upon cellular fractionation (such as ASF/SF2 or snRNP A1) showed any detectable relocalisation into the cytoplasm of intact cells (see Fig. 1D and data not shown). The increase seen by SILAC could either be below the detection limit for microscopy and/or reflect ‘leaking’ of proteins upon cellular fractionation as a result of their increased mobility.

**Fig. 4.**
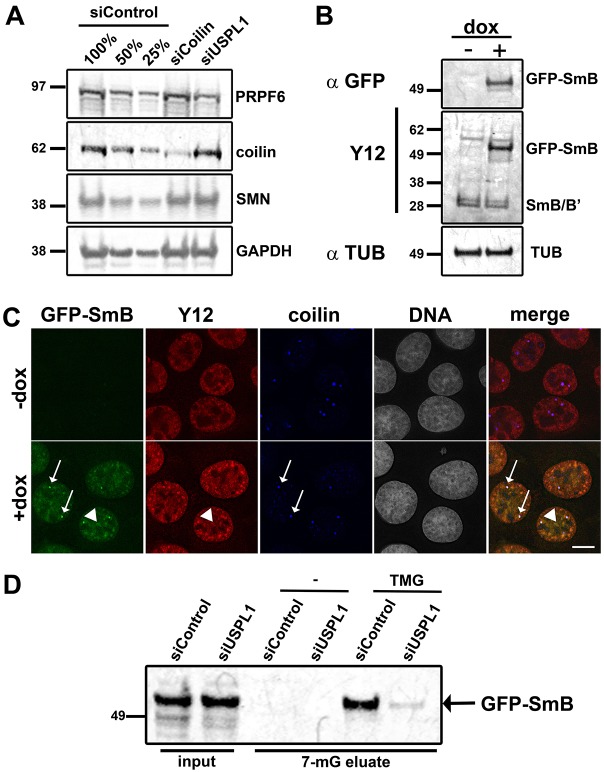
**Defects in snRNP production upon USPL1 knockdown.** (A) Total cell lysates of siRNA-treated U2OS cells were analysed by immunoblotting using antibodies as indicated. Different amounts of control lysate (100%, 50%, 25%) provide an internal standard for protein levels. Similar effects were observed with HeLa cells (data not shown). (B) Immunoblot of total cell lysate of U2OS GFP–SmB cells without (−dox) or 18 hours after (+dox) induction using the Y12 or an anti-GFP antibody showing expression of GFP–SmB at its expected molecular mass. Alpha-tubulin (TUB) stain serves as loading control. (C) U2OS GFP–SmB cells were analysed without (−dox) or 18 hours after (+dox) induction for expression and localisation of GFP-SmB by immunostaining using the Y12 and anti coilin antibody as marker for nuclear speckles (arrowheads) and Cajal bodies (arrows), respectively. Scale bar: 10 µm. (D) Formation of nascent snRNPs was analysed by TMG immunoprecipitation in siRNA-treated U2OS GFP–SmB cells. Empty Protein-G–agarose beads (-) serve as a control. Bound proteins were eluted from the beads using 25 mM 7-methylguanosine (7-mG) and analysed for the presence of GFP–SmB by immunoblotting for GFP. Input corresponds to 0.75% of the material used in the immunoprecipitation.

As shown in supplementary material Fig. S2A, a subset of proteins was reduced in overall protein levels upon USPL1 knockdown (∼2% of all identified proteins in the TLC; indicated by red box), including the U5 snRNP proteins PRPF6 and snRNP40, and the U1 snRNP protein snRNP70. These MS data were confirmed for PRPF6 by immunoblotting of total cell lysates obtained from USPL1-depleted U2OS cells (see Fig. 4A). The blotting data showed that PRPF6 protein levels were reduced by ∼50%, which is consistent with the SILAC data. Importantly, siRNA-mediated knockdown of coilin had no comparable effect, demonstrating that the changes in total protein levels were specific for knockdown of USPL1 (Fig. 4A). In comparison, levels of neither coilin nor SMN were affected by USPL1 knockdown, in agreement with earlier observations (supplementary material Fig. S1A). In conclusion, knockdown of USPL1 causes dramatic changes in the abundance and nucleocytoplasmic distribution of specific spliceosome components.

### Assembly and recycling of snRNPs are compromised upon knockdown of USPL1

We next investigated whether snRNP formation was affected by knockdown of USPL1. Considering that PRPF6 is required for the formation of the tri-snRNP complex (Makarov et al., 2000; Schaffert et al., 2004), we first investigated tri-snRNP formation. For this, we immunoprecipitated PRPF4, a component of the U4/6 di-snRNP, from nuclear extracts prepared from either control, coilin-knockdown or USPL1-knockdown HeLa cells, respectively. All immunoprecipitates were tested for the U5 snRNP components PRPF8, snRNP200 and EF-TUD, by immunoblot analysis (supplementary material Fig. S2C). All these proteins were reduced in the PRPF4 immunoprecipitates from USPL1-knockdown cells (right lane, supplementary material Fig. S2C), demonstrating a reduction in tri-snRNP formation. By contrast, little or no reduction in the amount of co-precipitated U5 snRNP proteins could be detected upon treatment with siRNA against coilin. This is consistent with previous reports showing that tri-snRNP formation still occurs in the nucleoplasm in the absence of coilin, albeit with reduced efficiency compared with Cajal bodies (Stanek and Neugebauer, 2004). This suggests that reduced tri-snRNP formation is a phenotype specifically associated with the knockdown of USPL1.

The altered localisation pattern for spliceosome components seen upon cellular fractionation in USPL1-knockdown cells might indicate either a reduction in complex assembly, and/or a change in the stability of snRNP complexes (Fig. 3D). We therefore analysed whether nascent snRNP production in general was affected upon knockdown of USPL1. It has been reported previously that Sm proteins that are N-terminally tagged with fluorescent proteins localise correctly and assemble into the heptameric Sm complex (Sleeman et al., 1998). We took advantage of this to distinguish newly assembled snRNPs, formed after we reduced levels of USPL1 using siRNA, from the pool of previously assembled snRNPs. Thus, we generated a U2OS cell line stably expressing GFP–SmB under a doxycycline (dox)-inducible promoter. When analysed by immunoblotting, a major band of the predicted size for GFP–SmB was detectable after 18 hours of dox induction (Fig. 4B). GFP–SmB localised correctly in Cajal bodies and nuclear speckles, as shown by immunostaining for coilin in combination with the Y12 anti-Sm antibody as markers for Cajal bodies and snRNPs, respectively (Fig. 4C). Equal amounts of protein lysate prepared from either control, or USPL1-knockdown cells after 18 hours of dox induction were subjected to immunoprecipitation with either empty beads (control), or beads pre-coupled to anti-TMG antibody, which recognises the 5′-terminal snRNA trimethyl cap structure. Precipitated snRNP complexes were eluted from the beads using 7-methylguanosine (7-mG) and GFP–SmB detected by immunoblotting (Fig. 4D). GFP–SmB was detected in TMG-eluates from control cells, but was reduced in cells transfected with siRNA against USPL1. This indicates that the production of nascent snRNPs is reduced upon knockdown of USPL1.

### USPL1 is involved in maintenance of snRNA levels

**Fig. 5.**
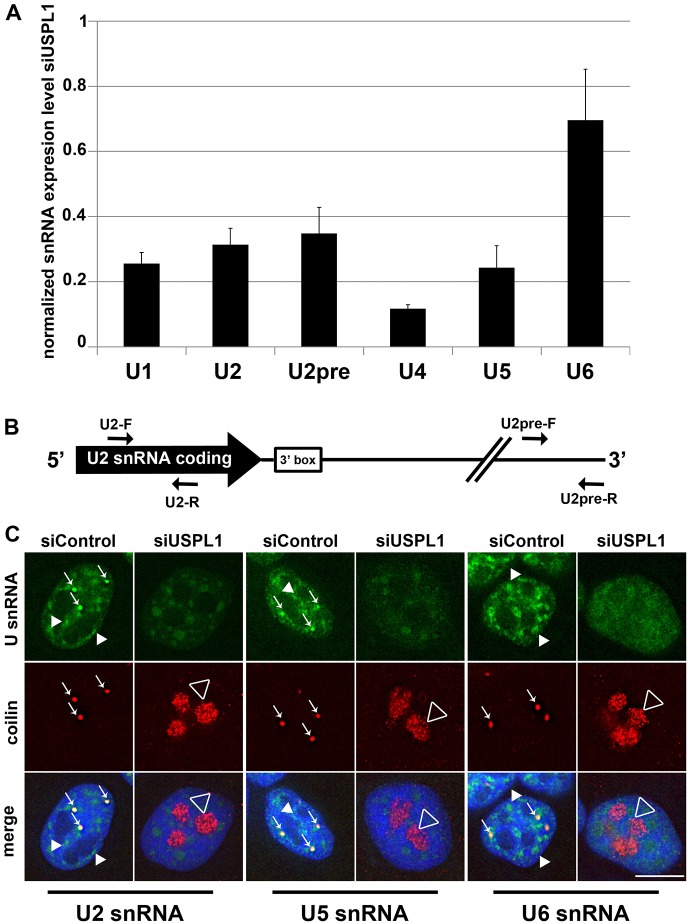
**Cellular snRNA levels are reduced upon knockdown of USPL1.** (A) qRT-PCR for different major U snRNA species in HeLa cells treated with siRNA against USPL1. U2pre represents the unprocessed U2 snRNA. Respective expression levels were normalised for levels of β-actin and snRNA levels of siControl-treated cells were set to 1. Bars represent the s.e.m. of four independent experiments, each measured in technical replicates of 2. (B) Schematic representation of the initial U2 snRNA transcript (adapted from Broome and Hebert, 2012) depicting additional 634 bp after the end of the U2 snRNA coding sequence and the position of the respective primers for qRT-PCR used for U2 snRNA in A. (C) HeLa cells treated with control siRNA or siRNA against USPL1 were subjected to RNA-FISH for U2, U5 and U6 snRNA using Alexa-Fluor-488-labelled probes. The immunostaining against coilin was used to monitor the efficiency of the siRNA treatment against USPL1. Cajal bodies are indicated by arrows, splicing speckles by arrowheads and nucleolar coilin by an open arrowhead. Scale bar: 10 µm.

After detecting a reduced assembly of snRNPs upon knockdown of USPL1, we wondered whether snRNA levels in general were affected. To address changes in snRNA expression upon USPL1 knockdown quantitatively, we performed qRT-PCR for the major snRNAs that are transcribed by either RNAPII or RNAPIII. In both HeLa and U2OS cells, primarily all RNAPII-transcribed snRNAs tested (U1, U2, U4 and U5 snRNA) exhibited reduced levels upon knockdown of USPL1 compared with control cells (Fig. 5A and supplementary material Fig. S3A). U1 and U2 snRNAs are transcribed as longer precursor snRNAs that get trimmed at their 3′ end by the Integrator complex before their export into the cytoplasm (Baillat et al., 2005; Chen and Wagner, 2010; Cuello et al., 1999; Egloff et al., 2008; Medlin et al., 2003). To differentiate between the possibility of USPL1 knockdown affecting either 3′ processing or snRNA transcription directly, we used primers specific for the 3′-precursor of U2 snRNA (Broome and Hebert, 2012) (U2pre, Fig. 5A,B and supplementary material Fig. S3A). Interestingly, the levels of this nascent transcribed form of the U2 snRNA were also reduced in a similar manner to the final processed form. This indicates that the transcription of snRNAs is compromised upon knockdown of USPL1.

In addition, we analysed snRNA localisation by RNA-FISH. This showed that snRNAs localise to nuclear speckles and Cajal bodies in control cells (Fig. 5C and supplementary material Fig. S3B, arrowheads and arrows, respectively). In agreement with our qRT-PCR data, the signal for RNAPII-transcribed snRNAs was reduced upon knockdown of USPL1. Moreover, it concentrated in enlarged nuclear speckles that were similar to the structures detected by immunostaining for ASF/SF2 and Sm proteins after knockdown of USPL1 (compare Fig. 5C and supplementary material Fig. S3B with Fig. 1D and supplementary material Fig. S1C).

### USPL1 interacts with LEC components involved in RNAPII-mediated snRNA transcription

**Fig. 6.**
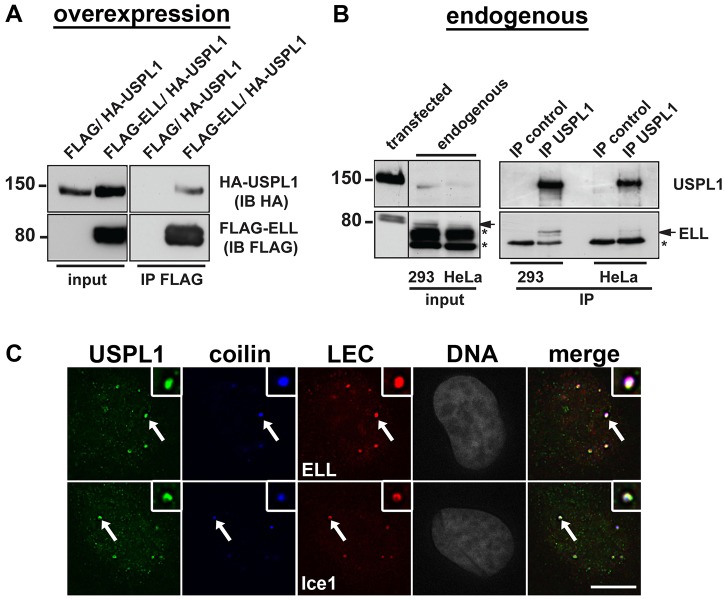
**USPL1 colocalises and interacts with members of the LEC complex.** (A) Total cell lysate obtained from HEK293 cells 24 hours after transfection with the indicated plasmids were subjected to immunoprecipitation using an anti-FLAG antibody. 1.5% of the total lysate was loaded as input (left). Bound proteins were analysed by immunoblotting using an anti-HA or anti-FLAG antibody (right). (B) Total cell lysate from either HEK293 (293) or HeLa cells was subjected to immunoprecipitation using the USPL1 antibody. Input corresponds to 1% of the total cell lysate (left) and a cell lysate sample of HEK293 cells transfected with either HA–USPL1 or FLAG–ELL was loaded onto the same gel as positive control for USPL1 or ELL, respectively. Empty Protein-G–agarose beads served as immunoprecipitation control. Bound proteins were analysed using either the USPL1 antibody or ELL antibody (right). The arrow indicates endogenous ELL, non-specific bands recognised by the ELL antibody are marked with asterisks. (C) Colocalisation of either ELL (top) or Ice1 (bottom) with USPL1 and coilin in Cajal bodies (arrows) shown by immunostaining of HeLa cells. Scale bar: 10 µm.

Previously, ELL and Ice1/KIAA0947 were identified in a large-scale screen for human de-ubiquitylating enzymes as two good candidate interactors for overexpressed USPL1 (Sowa et al., 2009). Both proteins have recently been shown to be involved in the regulation of snRNA transcription as components of the little elongation complex (LEC) in *Drosophila* and mammalian cells (Hu et al., 2013; Smith et al., 2011). To confirm the interaction of USPL1 with members of the LEC, we first used transient transfection of FLAG–ELL and HA–USPL1. As shown in Fig. 6A, HA–USPL1 was detected by protein immunoblotting following immunoprecipitation of FLAG–ELL, but was not detected following immunoprecipitation from extracts expressing the FLAG tag alone. We next employed affinity-purified antibodies against USPL1 to immunoprecipitate endogenous USPL1 from both HEK293 and HeLa cells. In both cell lines, we detected endogenous ELL by immunoblotting following immunoprecipitation of USPL1 (arrow, Fig. 6B), but not following the control immunoprecipitation.

**Fig. 7.**
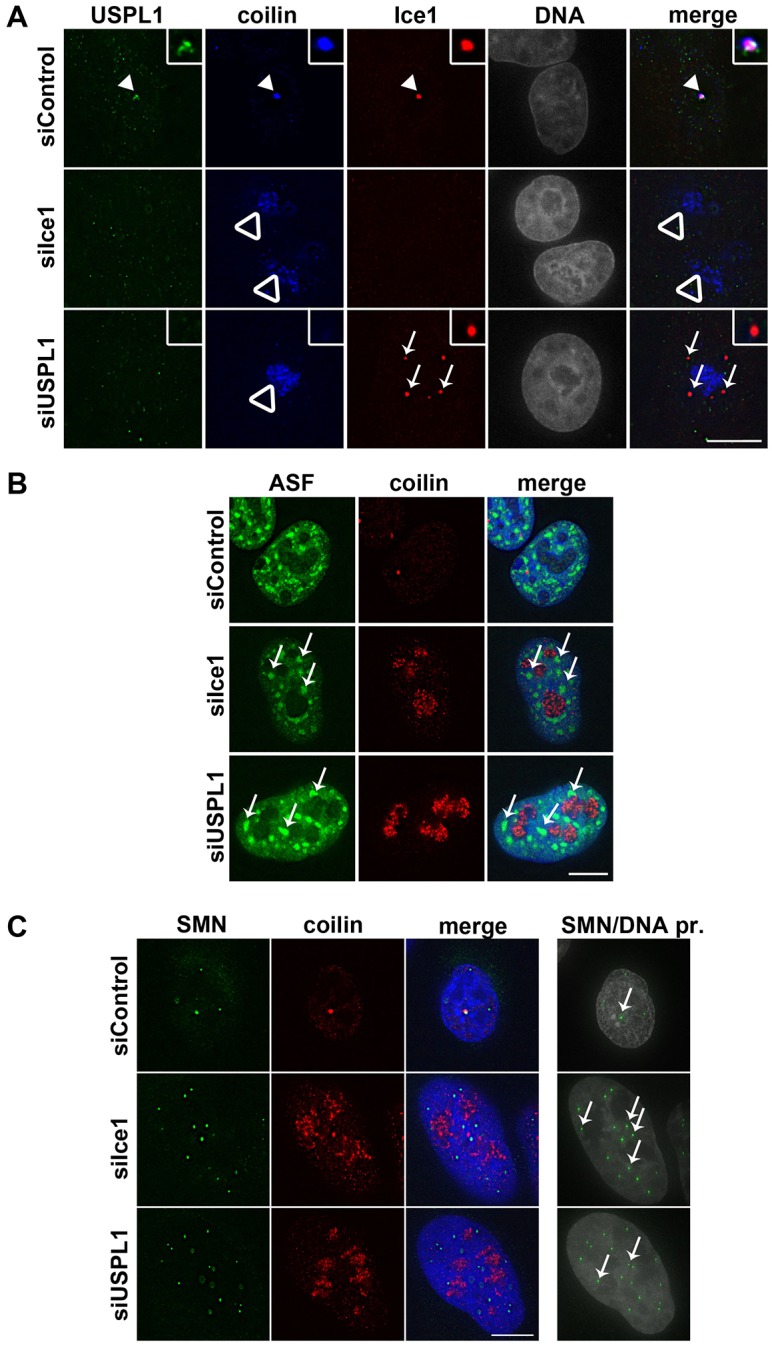
**Knockdown of Ice1 has comparable consequences for nuclear architecture as USPL1 knockdown.** (A) Immunofluorescence of HeLa cells transfected with siRNA as indicated were stained against USPL1, Ice1 and coilin. The arrowhead indicates a Cajal body in control cells with all three proteins present (top). Nucleolar coilin upon Ice1 or USPL1 knockdown is indicated by open arrowheads (middle and bottom, respectively). Arrows in the bottom panel highlight nuclear foci containing Ice1 in the absence of USPL1. (B) Immunofluorescence of siRNA treated U2OS cells against coilin and ASF. Arrows highlight enlarged, rounded nuclear speckles for ASF upon siIce1 (middle) or siUSPL1 (bottom) transfection. (C) Immunofluorescence of siRNA treated U2OS cells against coilin and SMN. A maximum-intensity projection illustrates the total number of SMN-containing nuclear foci (arrows in right panel). Scale bars, 10 µm.

USPL1, ELL and Ice1 have all been reported to localise to Cajal bodies (Hu et al., 2013; Polak et al., 2003; Schulz et al., 2012; Smith et al., 2011). As shown in Fig. 6C (arrows), both Ice1 and ELL colocalised with USPL1 in Cajal bodies. To address the possible interdependence of these proteins with respect to their recruitment to nuclear bodies, HeLa cells were transfected with either siRNA against Ice1, or against USPL1, and their localisation was analysed by immunostaining (Fig. 7A). As seen upon reduced levels of USPL1, knockdown of Ice1 resulted in disassembly of Cajal bodies and redistribution of coilin into the nucleolus (open arrowheads, Fig. 7A). We did not detect any remaining nuclear foci containing USPL1 after transfection of siRNAs against Ice1, suggesting that the recruitment of USPL1 into foci might depend on the presence of Ice1. However, knockdown of USPL1 did not lead to the disappearance of Ice1-containing nuclear foci, at least in HeLa cells. Instead, some HeLa cells showed an increase in the number of nuclear foci containing Ice1 (Fig. 7A, arrows in bottom panel). However, we were unable to detect persistent Ice1 nuclear foci after transfection of siRNA against USPL1 in U2OS cells.

Interestingly, enlarged, rounded nuclear speckles for ASF/SF2 (Fig. 7B arrows in middle panel) were detected upon efficient knockdown of Ice1, similar to those observed with USPL1 knockdown (Fig. 7B, arrows in bottom panel). Additionally, the number of nuclear SMN foci increased compared with that in control cells (Fig. 7C, arrows in projection). Therefore, knockdown of the LEC component Ice1 results in similar phenotypes regarding nuclear localisation of SMN and splicing speckles as seen following knockdown of USPL1.

### USPL1 is associated with U snRNA gene loci

**Fig. 8.**
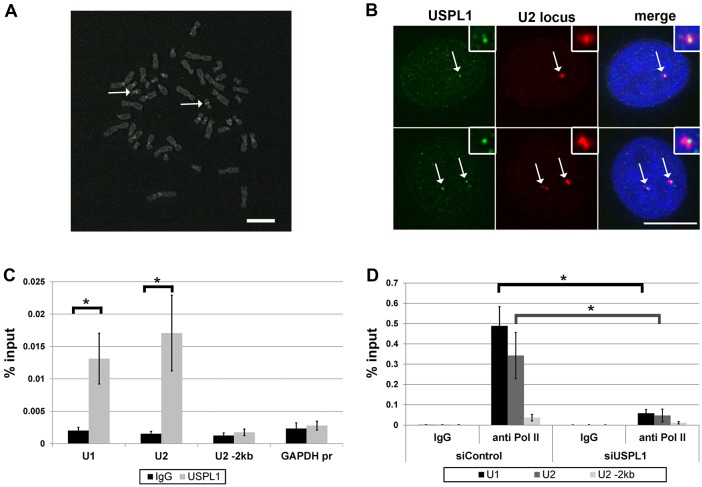
**USPL1 is associated with U snRNA gene loci by DNA-FISH and ChIP.** (A) Specificity of the probe for the U2 gene array (Cy5, shown in green; arrows) demonstrated on a metaphase spread from normal human male lymphocytes. (B) HeLa cells were subjected to DNA-FISH for the U2 gene locus (Cy5, shown in red) in combination with immunostaining for USPL1 (Alexa Fluor 488, shown in green). Arrows indicate nuclear foci containing both USPL1 and the U2 gene locus. Scale bars: 10 µm. (C) ChIP from HeLa cells using the USPL1 antibody is displayed in comparison to IgG control for the respective gene locus (U1, U2 or GAPDH). Bars represent the s.e.m. of six independent experiments, each analysed as technical replicate of two in the qPCR-reaction. Statistical significance was determined using an unpaired, heteroscedastic Student's *t*-test. (D) RNAPII occupancy detected by ChIP against the CTD of RNAPII (4H8 antibody) in comparison to IgG control for the respective gene region (U1, U2, U2 −2 kb) upon USPL1 knockdown. Bars represent the s.e.m. of four independent experiments, each analysed as technical replicate of two in the qPCR reaction. Statistical significance was determined using an unpaired, heteroscedastic Student's *t*-test. *P<0.05.

Components of the LEC have been shown to be enriched on RNAPII-transcribed snRNA genes (Hu et al., 2013; Smith et al., 2011). Our findings, together with other data (Sowa et al., 2009), indicate an association of USPL1 with components of the LEC. Therefore, we decided to investigate whether USPL1 itself could be found associated with snRNA gene loci. Previously, Cajal bodies defined by the marker protein coilin were shown to be associated with several snRNA gene loci by DNA-FISH (Frey and Matera, 1995; Gao et al., 1997; Jacobs et al., 1999; Smith et al., 1995). The U2 genes are located in a 120 kb cluster of ∼6 kb long repeat units on the q-arm of chromosome 17 (Lindgren et al., 1985; Van Arsdell and Weiner, 1984; Westin et al., 1984). We designed a set of fluorescently labelled FISH probes consisting of four individual fragments, covering the entire U2 gene locus. The probes hybridised with exactly two loci on two different chromosomes on metaphase spreads of normal human male lymphocytes, which is consistent with specificity for the U2 gene locus (Fig. 8A, arrows). When tested on HeLa interphase cells, the U2 FISH probe frequently associated with Cajal bodies, as judged by co-staining for coilin (data not shown), consistent with previous reports (Frey and Matera, 1995; Smith et al., 1995). When we performed U2 DNA-FISH on HeLa cells in conjunction with immunostaining for USPL1, nuclear foci containing USPL1 were detected in close association with the FISH signal for the U2 gene locus (Fig. 8B, arrows).

As a complementary approach to test for association of USPL1 with U snRNA genes, we used chromatin immunoprecipitation (ChIP) (Fig. 8C). USPL1 protein was precipitated from formaldehyde-crosslinked HeLa cells and associated DNA was tested for the presence of U snRNA gene sequences using U1 and U2 specific primers. We detected a significant enrichment for the U1 and U2 gene regions using the USPL1 antibody compared with the IgG control. By contrast, no significant enrichment for either the GAPDH gene promoter, or a region 2000 bp upstream of the U2 promoter (U2, −2 kb), was detected (Fig. 8C). To further test whether USPL1 is directly involved in RNAPII-mediated snRNA transcription, we performed ChIP for RNAPII upon knockdown of USPL1. The results obtained by using two different antibodies clearly show a significant reduction in RNAPII occupancy at the U1 and U2 gene regions in cells treated with siRNA against USPL1 (Fig. 8D and supplementary material Fig. S4). We conclude that USPL1 is required for RNAPII-dependent snRNA transcription and is a component of complexes that can bind to U snRNA genes.

## DISCUSSION

In this study we have presented data investigating a functional role for the Cajal-body-associated, SUMO isopeptidase USPL1. Using a combination of fluorescence microscopy, molecular biology and proteomic approaches, we demonstrate that USPL1 interacts with components of the RNAPII-associated LEC and is associated with the U1 and U2 snRNA gene loci. Efficient knockdown of USPL1 by RNAi leads to reduced RNAPII-mediated snRNA transcription, diminished production of snRNPs and altered pre-mRNA splicing. Our data suggest that USPL1 is involved in snRNA transcription and support the view that Cajal bodies have a role in snRNP biogenesis.

By using RNAi in combination with cellular fractionation and MS-based protein analysis we detected a striking effect of reduced USPL1 levels on proteins associated with snRNP biogenesis and/or pre-mRNA splicing. This was supported by our accompanying immunofluorescence studies in cells with reduced levels of USPL1, showing changes for Cajal bodies and splicing speckles, but not for either PML bodies, or the nucleolus. We observed a decrease in RNAPII-transcribed snRNA levels upon USPL1 knockdown and subsequent defects in snRNP production. Although it is transcribed by RNAPIII, U6 snRNA levels were also slightly affected by knockdown of USPL1. However, it is known that U6 snRNA assembles into a U4/U6 di-snRNP complex with the RNAPII transcribed U4 snRNA and non-incorporated U6 snRNA has a higher turnover rate than the assembled form (Sauterer et al., 1988). A link between RNAPIII-mediated U6 transcription and RNAPII has also been suggested (Listerman et al., 2007). The disruption of snRNP biogenesis probably explains both the small effect of USPL1 knockdown on U6 snRNA levels and the increase of snRNP proteins in the cytoplasm detected upon USPL1 knockdown by our cellular fractionation study (Fig. 3C). This increase could either reflect a ‘leaking’ of nuclear proteins into the cytoplasm during the cell fractionation procedure and/or proteins that under normal conditions are imported into the nucleus only in complex with their respective snRNA.

Interestingly, we also detected changes in the pattern of specific pre-mRNA splicing after USPL1 knockdown. Previously, defects in splicing and the composition of the pool of snRNA species have been reported upon SMN deficiency, depending on the severity of the SMN reduction (Boulisfane et al., 2011; Campion et al., 2010; Gabanella et al., 2007; Gao et al., 1997; Zhang et al., 2008). However, we found that SMN levels were either not, or only mildly, affected by knockdown of USPL1, indicating that a distinct mechanism is involved. This raises the intriguing possibility that splicing could be regulated *in vivo*, either during development, or in distinct human tissues, by varying levels of USPL1.

Transcription of snRNA genes by RNAPII differs significantly from the transcription of most protein-coding genes (for recent reviews see Egloff et al., 2008; Jawdekar and Henry, 2008). However, the mechanism of snRNA transcription is still not understood in detail. Most recently, Shilatifard and colleagues showed by a genome-wide ChIP and RNA-sequencing approach that ELL and Ice1/2, as part of the LEC, are enriched at RNAPII snRNA genes and that Ice1 and ELL are required for initiation and elongation of snRNA expression, respectively (Hu et al., 2013; Smith et al., 2011).

Here, we show that human USPL1 not only colocalises and interacts with components of the LEC, but also demonstrate that endogenous USPL1 is present at snRNA gene loci in human cells. Our interaction data are supported by the findings of Harper and colleagues, who identified human ELL and Ice1 in a large-scale proteomic approach upon USPL1 overexpression (Sowa et al., 2009). Importantly, knockdown of either USPL1, or the LEC member Ice1, causes similar phenotypes regarding decreased snRNA levels (compare our data with Hu et al., 2013; Smith et al., 2011) and nuclear architecture (Fig. 7A–C). This suggests that USPL1, similar to the LEC, has an important role in snRNP biogenesis at the transcriptional level. This is supported by our data showing a significantly reduced association of RNAPII with snRNA gene loci upon USPL1 knockdown. We therefore speculate that USPL1 might either be a component of the LEC, or else is functionally associated with it. Future studies will show whether USPL1 is involved in transcription initiation and/or elongation. In *Drosophila*, CG8229, a protein with limited homology to the N-terminus of USPL1, was identified as a binding partner for Ice1 and Ice2 in *Drosophila* (Smith et al., 2011), but it lacks a catalytic domain. Therefore it will be interesting to analyse whether and to what extent the desumoylating activity of USPL1 contributes to snRNA transcription in human cells.

## MATERIALS AND METHODS

### Plasmids

To generate GFP–SmB, the coding sequences for GFP and SmB were PCR amplified and inserted into the pcDNA5-FRT/TO vector (Invitrogen) via *Hin*dIII/*Kpn*I and *Kpn*I/*Not*I sites, respectively. For the generation of FLAG–ELL, ELL was amplified from pCMV-ELL (Imagene) and inserted using *Bam*HI/*Eco*RI sites into pcDNA3.1 (Meulmeester et al., 2008).

### Cell culture, transfection and metabolic labelling with 5-ethynyl uridine

Clonal U2OS GFP–SmB cells were selected and maintained using 150 µg/ml hygromcin B and 15 µg/ml blasticidin-HCl. Expression of GFP–SmB was induced with 10 ng/ml doxycyclin for 18 hours. Transfection of DNA was performed using Effectene (Qiagen), Jet Prime (Polyplus transfection) or polyethylenimine (PEI) (Durocher et al., 2002). siRNA transfections (20 nM final concentration; see supplementary material Table S4 for siRNA sequences) were performed using Lipofectamine RNAiMaxx (Invitrogen) omitting antibiotics and experiments were analysed after 2 days. For the siRNA transfection in SILAC medium, OptiMem was replaced with the respective SILAC medium lacking serum and antibiotics. 5-ethynyl uridine (EU) labelling was performed using the EU-Click-iT kit (Invitrogen).

### Antibodies

A list of all primary antibodies used in this study can be found in supplementary material Table S3. Except for goat anti-chicken FITC (Sigma, F-8888), Alexa Fluor 488, Alexa Fluor 594 or Alexa Fluor 647 dye-conjugated secondary antibodies from donkey (Molecular Probes) were used for immunofluorescence. For immunoblotting, either a Li-Cor Odyssey CLx (Li-Cor Biosciences) in combination with goat secondary antibodies conjugated to Alexa Fluor 680 or Alexa Fluor 800 (Tebu-Bio/Invitrogen) (Fig. 4 and supplementary material Fig. S2C) or HRP-conjugated secondary antibodies (Jackson Immunoresearch) with ECL detection (Thermo) (Fig. 6A,B; supplementary material Fig. S1A) were used.

### Immunofluorescence and fluorescence microscopy

Immunofluorescence and image acquisition was essentially performed as described (Hutten et al., 2011) except that 1% normal donkey serum was used as blocking buffer. Images were acquired with a DeltaVision Core Restoration microscope (Applied Precision) mounted on an Olympus IX71 stand with a 60× /1.42 NA oil immersion objective lens using 1×1 bin with a section spacing of 0.2 µm. Exposure time was set to provide an intensity of at least ∼1000 counts on a 12-bit Coolsnap HQ2 camera at gain 4 (Roper) for control cells. Note, that for reasons of visibility of nucleolar coilin, longer exposure times were used and different image processing applied on siRNA-treated cells compared with control cells. Coilin-knockdown cells were treated identically to cells transfected with siIce1 or siUSPL1 for image acquisition and processing. Images were corrected by flat field calibration, deconvolved and corrected for chromatic aberration using SoftWorx (Applied Precision). Processing and image analysis was performed using SoftWorx and Adobe Photoshop/Illustrator. Unless stated otherwise, a single *z*-stack is shown in the figures. Maximum intensity projections were adjusted differently than corresponding single *z*-sections for clarity of the staining.

For supplementary material Fig. S1C, cells were fixed using 3.7% formaldehyde, permeabilised for 10 minutes in 0.2% Triton X-100 on ice and blocked in 3% BSA, 0.1% Tween-20 in PBS for 1 hour. Antibody incubation was performed in blocking buffer for 1.5 hours at room temperature. Cells were mounted in mounting medium (Dako) and images acquired using an Axioskop (Observer zl, Zeiss) and an Axiocam Mrm camera (Zeiss).

### RNA and DNA fluorescence in-situ hybridisation (FISH)

#### RNA-FISH

The protocol for FISH against U snRNAs was adapted from Taneja and colleagues (Taneja et al., 1992) with the following modifications: cells were fixed and permeabilised as described above for immunofluorescence. The hybridisation buffer contained 0.02% BSA and additional 0.4 mM ribovanadyl complex. Probes were obtained as 5′ Alexa Fluor 488 derivatives (sequences according to Schaffert et al., 2004) (supplementary material Table S8). After hybridisation, cells were washed with 0.5% Triton X-100 in PBS, re-fixed with paraformaldehyde and subjected to immunofluorescence.

#### DNA-FISH

A pool of four different, Cy5-dCytosine nick-labelled (nick-translation kit; GE Healthcare), 1.5–2 kb long PCR-products (see supplementary material Table S9) covering the whole 6 kb U2 gene locus was used as a probe. The labelled probe (∼130 ng) was ethanol precipitated in the presence of sodium acetate and human Cot-1 DNA and resuspended in hybridisation buffer (Hybrisol, Abbot Molecular). Before hybridisation, cells grown on microscopy glass slides were subjected to immunofluorescence using antibodies twice (primary) to four times (secondary) as concentrated, re-fixed and dehydrated in 70%, 90% and absolute ethanol. Dehydration was repeated after denaturing genomic DNA for 5 minutes in 2× SSC, 70% formamide at 80°C in an adaptation of published methods (Matera et al., 1995). The probe was prewarmed to 37°C, added to air-dried, prewarmed slides, denatured for 2 minutes at 80°C and hybridisation was performed overnight in a humidified chamber at 37°C. After successive washing steps in 2× SSC, 50% formamide (5 minutes at 40°C) and 1× SSC (three times for 5 minutes at room temperature), cells were briefly rinsed in PBS and nuclei were counterstained with Hoechst 33342 (Sigma) before mounting as above. Metaphase spreads of normal human male lymphocytes were obtained from Abbot Molecular (Maidenhead, UK).

### Non-quantitative and real-time quantitative RT-PCR (qRT-PCR)

Total RNA was isolated from siRNA-treated cells using either the miRNeasy kit (Qiagen; qRT-PCR) or NucleoSpin RNA kit (Macherey and Nagel; qualitative RT-PCR) including a DNase digest. Equal amounts of RNA from control or USPL1-knockdown cells were subjected to qualitative RT-PCR using the 1-Step RT-PCR kit from Qiagen with gene-specific primers (see supplementary material Table S5) and analysed by ethidium bromide agarose gel electrophoresis. qRT-PCR was performed on 0.4 ng of RNA per reaction (or 4 ng RNA in the case of U2pre-snRNA) in duplicate using the QuantiFast RT-PCR SYBR-Green Mix (Qiagen) in a Roche Light Cycler 480. Primers (see supplementary material Table S6) were tested for their PCR efficiency and the formation of primer dimers under qRT-PCR conditions.

### Chromatin immunoprecipitation (ChIP)

ChIP using the RNAPII and USPL1 antibody was performed as described (Yin et al., 2012) using ∼1–2×10^7^ HeLa cells. ChIP for USPL1 was performed using 1 µg of antibody in the presence of 0.3% Brij-35 overnight. For comparison of RNAPII gene locus occupancy in siRNA-treated cells, total protein concentration was determined by a bicinchoninic acid assay (Thermo Scientific) and a SpectraMax MSe (Molecular Devices) and equal amounts of total cell lysate were subjected to ChIP using either 1 µg (mouse) or 2 µg (rabbit) anti RNAPII antibody in the presence of 0.1% Brij-35 overnight. In general, 10% of the lysate was retained as input, the rest was split for incubation with either control IgG (Goat, Santa Cruz Biotechnology, rabbit/mouse, Jackson ImmunoResearch) or USPL1/RNAPII antibody. Antibodies were captured using Protein-G dynabeads, After RNase treatment, reversal of crosslinks and protein K digestion (125 µg/ml) of input and eluates, DNA was purified using the PCR purification kit (Qiagen) and enriched sequences analysed by qPCR using a Roche 480 Light cycler and the Quantitect SYBR-Green PCR mix (Qiagen) (for primers, see supplementary material Table S7). For quantification, crossing point (Cp) values were calculated using the absolute quantification analysis/fit points method (Light cycler 480 Software module) and ChIP values were normalised for the input for each primer pair.

### RNA and protein co-immunoprecipitations

For the TMG-immunoprecipitation, siRNA-transfected U2OS cells (∼1–2×10^7^ cells) were harvested 18 hours after induction of GFP–SmB expression and lysed in RIPA buffer [50 mM Tris-HCl, pH 7.5, 150 mM NaCl, 1% NP40, 0.5% deoxycholate and protease inhibitors (Roche)]. The lysate was sonicated and cleared by centrifugation at 17,000 ***g***. Equal amounts of protein lysate from control or USPL1-depleted cells were incubated overnight with 30 µl (slurry) TMG-agarose (Calbiochem) or Protein-G agarose beads as control. After several washes, bound snRNP complexes were eluted using 25 mM 7-methylguanosine in PBS (Sigma) and analysed by immunoblotting for the presence of newly incorporated GFP–SmB.

For immunoprecipitation of tri-snRNP complexes, nuclear extracts from ∼2–6×10^7^ siRNA-transfected HeLa cells were prepared as follows: Cells were harvested and incubated in one packed cell volume (PCV) of NE1 buffer (10 mM HEPES, pH 7.6, 1.5 mM MgCl_2_, 10 mM KCl, 1 mM DTT) for 15 minutes on ice. Afterwards, cells were sheared using a 23 gauge needle and the nuclei extracted by incubation in 2/3 PCV of NE2 buffer (20 mM HEPES, pH 7.6, 1.5 mM MgCl_2_, 25% glycerol, 420 mM NaCl, 0.2 mM EDTA, 1 mM DTT, 0.5 mM PMSF) for 30 minutes at 4°C. Nuclear extracts were cleared by centrifugation at 17,000 ***g***. 250–500 µg of nuclear extract was diluted 10- to 16-fold in IPP150 buffer (10 mM Tris-HCl, pH 7.5, 150 mM KCl, 0.1% NP40) (Blencowe et al., 1993) and subjected to immunoprecipitation using 2 µg of control rabbit IgG (Jackson ImmunoResearch) or anti Prp4 antibody (HPA/Sigma) overnight. Antibodies were captured using Protein-G dynabeads (Invitrogen) and bound proteins were eluted using 2× LDS sample buffer (Invitrogen) and analysed by immunoblotting.

For immunoprecipitation of FLAG–ELL and USPL1, HeLa or HEK293 cells were harvested in lysis buffer (50 mM Tris-HCl, pH 7.5, 150 mM NaCl, 1% NP40, 5 mM EDTA, 5 mM EGTA, 1 mM DTT with protease inhibitors) for 30 minutes on ice. Cells were sheared using a 21 gauge needle and the extracts were cleared by centrifugation at 10,000 ***g***. 5 mg of lysate was pre-cleared and subjected to immunoprecipitation for 3 hours using beads pre-blocked with 1% ovalbumin protein. Beads were either loaded with 5 µg anti-FLAG (for the transfected FLAG and FLAG–ELL) or anti-USPL1 antibodies (for endogenous USPL1). Empty Protein-G–agarose beads (Roche) served as control in the immunoprecipitation of endogenous USPL1. After several washes, bound proteins were eluted using 2× SDS sample buffer and analysed by immunoblotting.

### Cellular fractionation, SILAC and LC-MS/MS

HeLa cells were fully metabolically labelled by growing for at least six doublings in lysine- and arginine-deficient DMEM SILAC-medium (Fisher) supplemented with dialysed FBS, 100 U/ml penicillin and 100 µg/ml streptomycin in addition to labelled amino acids (42 µg/ml arginine and 73 µg/ml lysine; Cambridge Isotope Lab) as follows: R_0_K_0_ (L-arginine and L-lysine) or R_10_K_8_ (L-arginine ^13^C/^15^N and lysine ^13^C/^15^N). Two days after siRNA transfection, an equal number of cells were mixed and cytoplasmic, nucleoplasmic and nucleolar fractions prepared as described (Andersen et al., 2002; Boisvert et al., 2010). The cytoplasmic fraction was cleared from cellular debris by centrifugation at 9600 ***g*** at 4°C. Lysates were prepared by adding RIPA buffer (final concentration: 50 mM Tris-HCl, pH 7.5, 150 mM NaCl, 1% NP40, 0.5% sodium-deoxycholate with protease inhibitors) to the individual fractions. Protein concentration was determined by a bicinchoninic acid assay (Thermo Scientific) and a SpectraMax MSe (Molecular Devices). 20 µg protein of each fraction in loading buffer were separated by one-dimensional SDS-PAGE (4–12% Bis-Tris Novex mini-gel) and visualised using colloidal Coomassie Blue staining (Novex, Invitrogen). The individual gel lanes were cut into eight slices as indicated (Fig. 3A), destained, reduced with 10 mM DTT and alkylated in 50 mM iodacetamide prior to in-gel trypsin digest (Shevchenko et al., 1996). Tryptic peptides were extracted using equal volumes of 5% formic acid and acetonitrile, dried in a speedvac and resuspended in 5% formic acid. Peptides were analysed by LC/MS-MS on a Orbitrap Velos mass spectrometer over a 156 minute gradient (Thermo Fisher Sc.) and data analysed with MaxQuant (version 1.2.2.5) (Cox and Mann, 2008; Cox et al., 2011; Ong et al., 2002; Ong and Mann, 2007) and the Human UniProtKB and TrEmbl Database (retrieval date December 2011). Carbamidomethylation was set as a fixed modification, and oxidation of methionine, N-acetyl protein, glutamine to glutamic acid conversion and deamidation were searched as variable modifications. The match-between runs function was enabled and the maximum false discovery rate set to 1%. Those protein identifications that were derived from the decoy database, listed as common contaminant by MaxQuant or those only identified by a modification site were excluded from further data analysis. The mean of the log_2_ of the normalised H/L ratio was calculated based only on protein quantification data with >1 unique peptide for an individual fraction. In order to identify outliers, the arbitrary threshold was defined as twofold standard deviation (2σ) of the mean.

Protein enrichment analysis was performed using the Bioinfomatics database DAVID (v. 6.7) (Huang et al., 2009a; Huang et al., 2009b). Protein groups significantly enriched according to the MaxQuant analysis (>2σ) in either cytoplasm or nucleolus were analysed by functional annotation analysis against the background of all proteins identified with >1 unique peptide in the respective fraction.

## Supplementary Material

Supplementary Material
