# Supplementary material for: A role for the Cajal-body-associated SUMO isopeptidase USPL1 in snRNA transcription mediated by RNA polymerase II
Source: J Cell Sci. 2014 Mar 1;127(5):1065–78. doi: 10.1242/jcs.141788 (PMC3937775; doi:10.1242/jcs.141788)
Supplement: Supplementary Material [file supp_127_5_1065__index.html]

A role for the Cajal-body-associated SUMO isopeptidase USPL1 in snRNA transcription mediated by RNA polymerase II — Supplementary Material 

# A role for the Cajal-body-associated SUMO isopeptidase USPL1 in snRNA transcription mediated by RNA polymerase II

## JCS141788 Supplementary Material

**Files in this Data Supplement:**

- **Supplementary Material**
