# Supplementary material for: A role for the Cajal-body-associated SUMO isopeptidase USPL1 in snRNA transcription mediated by RNA polymerase II
Source: J Cell Sci. 2014 Mar 1;127(5):1065–78. doi: 10.1242/jcs.141788 (PMC3937775; doi:10.1242/jcs.141788)
Supplement: Supplementary Material [file supp_127.5.1065_JCS141788.pdf]

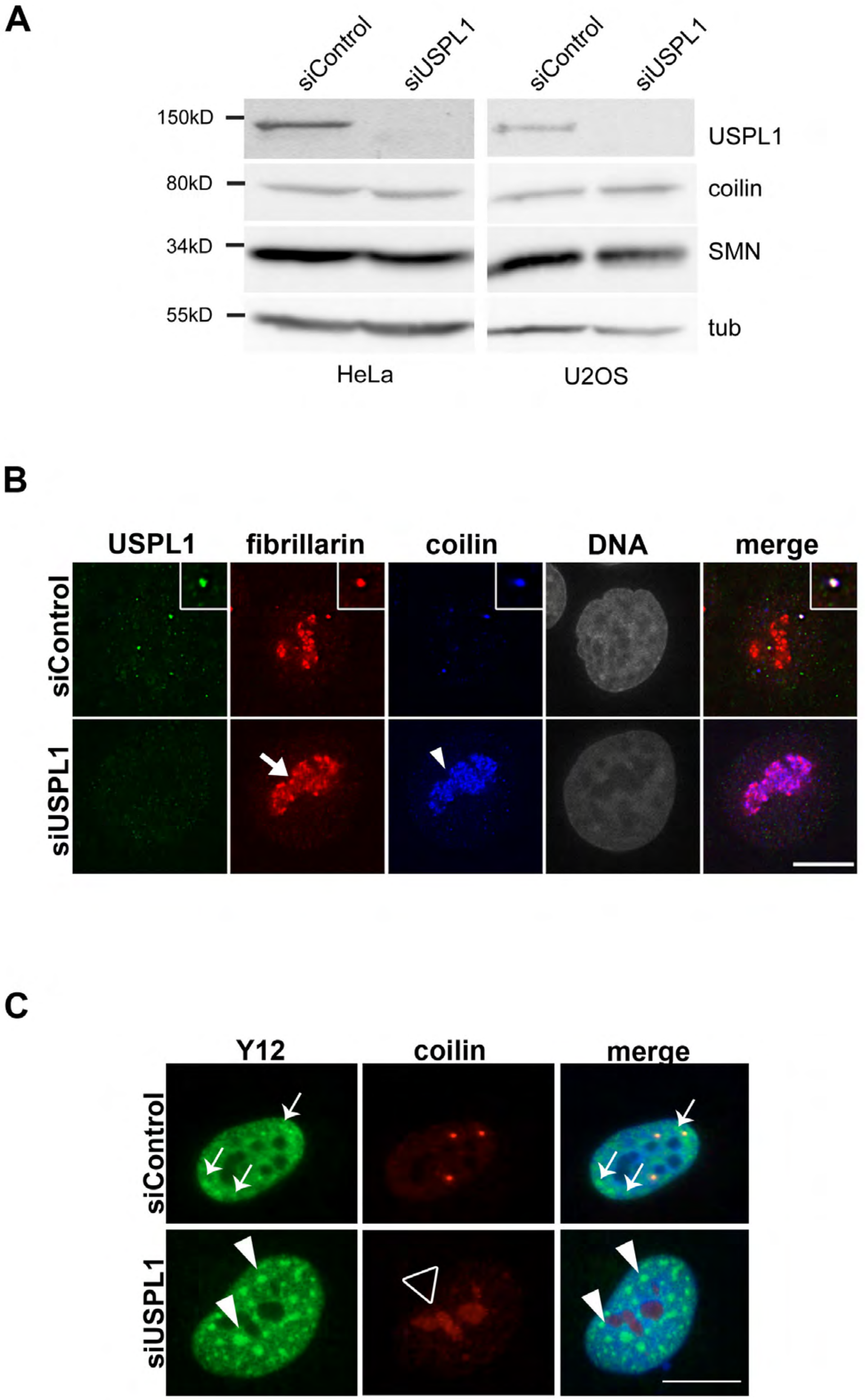

Fig. S1. USPL1 knockdown affects factors associated with pre-mRNA splicing. (A) Immunoblot for USPL1, coilin and SMN on total cell lysate obtained from HeLa and U2OS cells upon control- or USPL1-knockdown. Alpha tubulin stain serves as loading control. (B) Immunofluorescence of siRNA treated HeLa cells against fibrillarin, USPL1 and coilin. In a subset of cells, fibrillarin appears less condensed in the nucleolus upon USPL1 knockdown (indicated by arrow). Bar, 10 ∀m. (C) Immunofluorescence of siRNA treated HeLa cells against coilin and Sm proteins (Y12 antibody). Arrows highlight splicing speckles in control cells (top panel), arrowheads enlarged nuclear speckles for Sm proteins upon USPL1 knockdown. An open arrowhead highlights nucleolar coilin upon siUSPL1. Bar, 10 µm.

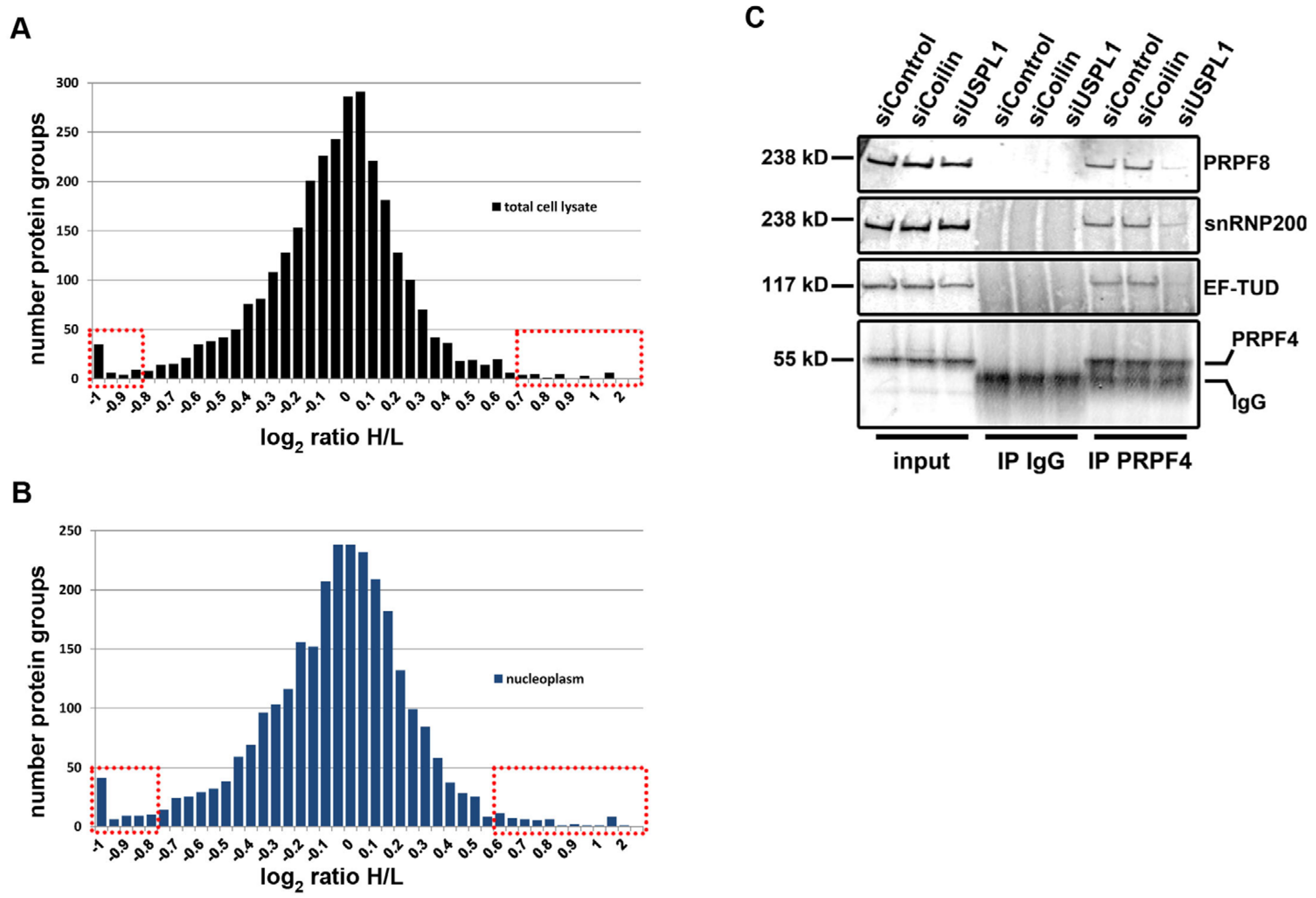

Fig. S2. Effects of USPL1 knockdown on the cellular proteome and tri-snRNP formation. (A,B) SILAC analysis of total cell lysate and nucleoplasm upon fractionation of HeLa cells and USPL1-knockdown. Only hits identified with at least 2 peptides in an individual fraction are displayed here. The $\log_2$ ratio heavy (H; siUSPL1)/light (L; siControl) is displayed as frequency histogram for total cell lysate (A) and nucleoplasm (B). Proteins groups above/below the arbitrary threshold are indicated by red lined boxes. (C) Control- (IgG) or PRPF4-immunoprecipitations (IP) of nuclear extracts prepared from siRNA treated HeLa cells were analyzed by immunoblot analysis with antibodies as indicated. Input corresponds to ~3% of nuclear extract used in the IP.

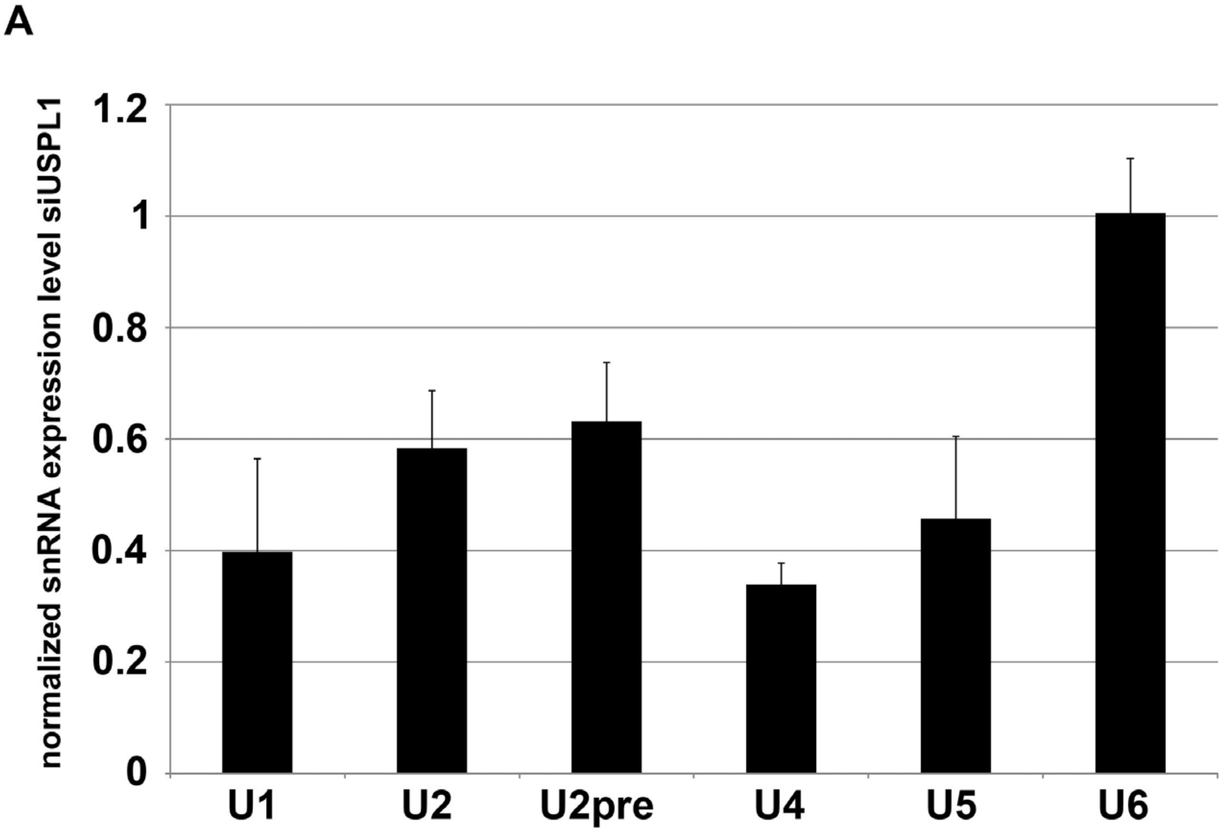

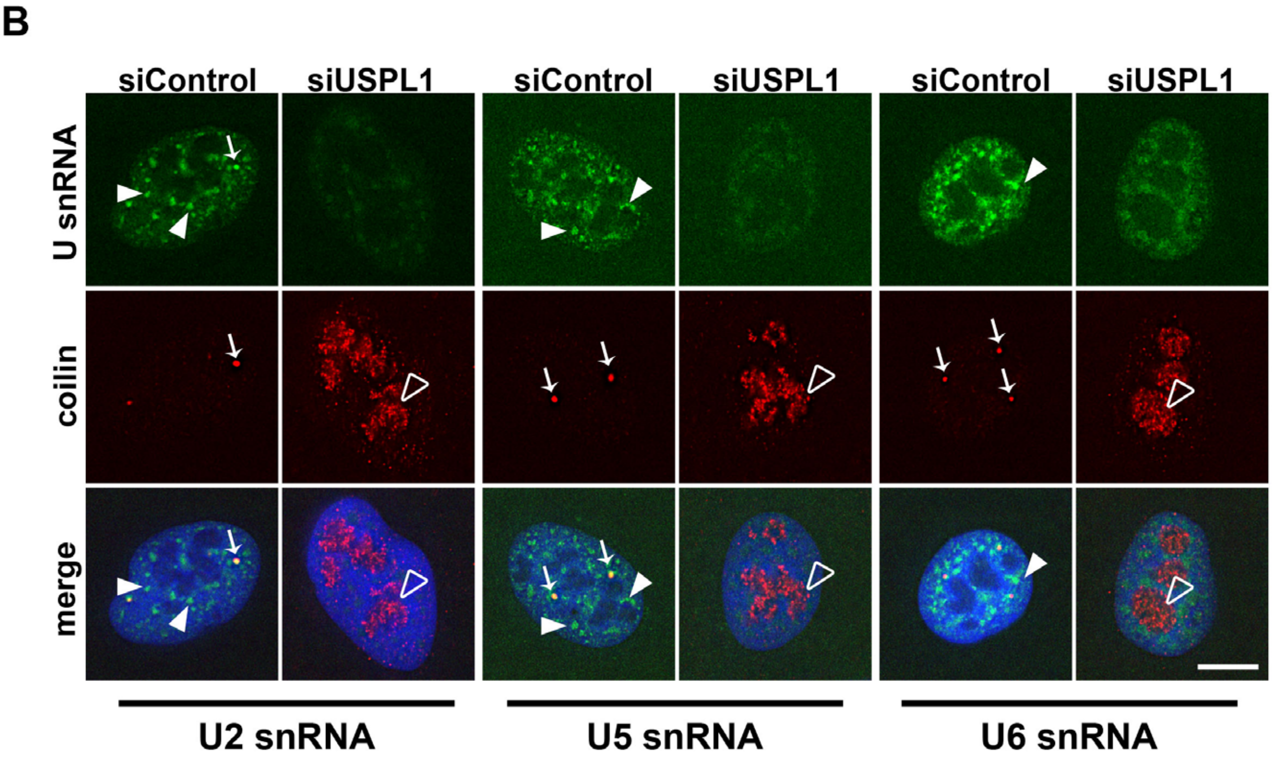

Fig. S3. USPL1 knockdown affects U snRNA transcription by RNAPII. (A) qRT-PCR for different U snRNA species in U2OS cells upon knockdown of USPL1. U2pre represents the unprocessed U2 snRNA. Respective expression levels were normalized for β–actin and snRNA levels of siControl treated cells were set to 1. Bars represent the standard error of 4 independent experiments, each measured in technical replicates of 2. (B) Control- or USPL1-depleted U2OS cells were subjected to RNA-FISH for U2, U5 and U6 snRNA using Alexa488-labeled probes. The immunostaining against coilin serves as control for the efficiency of the siRNA treatment against USPL1. CBs are indicated by arrows, splicing speckles by arrowheads and nucleolar coilin by an open arrowhead. Bar, 10 µm.

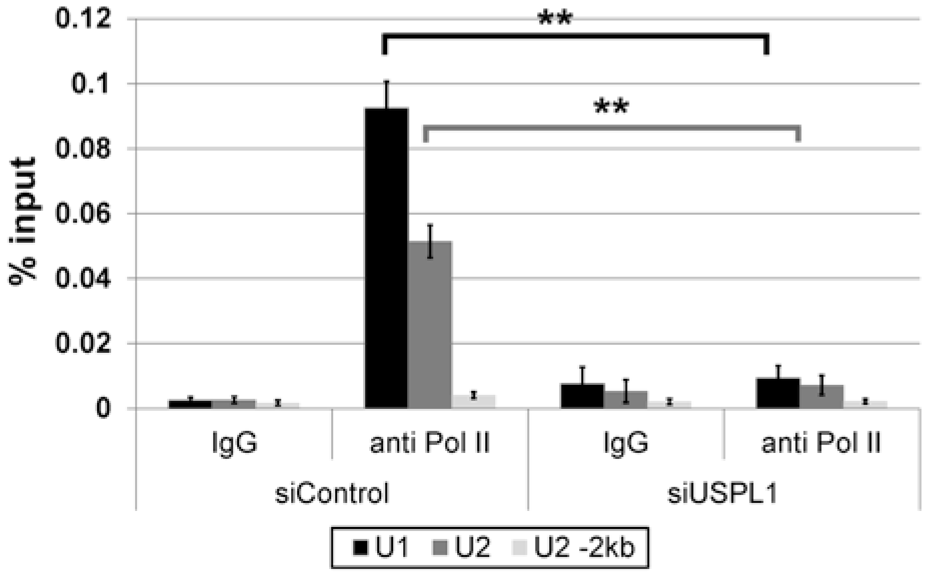

Fig. S4. Reduced RNAPII gene occupancy at snRNA loci. RNAPII occupancy detected by ChIP using an antibody against RNAPII (N20; sc-899) is significantly reduced at U1 and U2 snRNA gene loci upon USPL1 knockdown. Bars represent the s.e.m. of 4 independent experiments, each analyzed as technical replicate of 2 in the qPCR-reaction. Statistical significance was determined using an unpaired, heteroscedastic Student's t-test.

Supplementary Table S1. The first sheet contains protein group table with the total experimental dataset of the SILAC-MS based cellular fractionation upon USPL1 knockdown. T=TCL; C=cytoplasm; np=nucleoplasm; nol=nucleolus. The second sheet lists all splicing factors identified in SILAC-MS dataset with >1 peptide according to (Hegele et al., 2012).

Download Table S1

Supplementary Table S2. Result of DAVID analysis for proteins identified with >1 peptide in cytoplasmic and nucleolar fraction of SILAC-MS dataset.

Download Table S2

Supplementary Table S3. Antibodies

Download Table S3

Supplementary Table S4. siRNA sequences

Download Table S4

Supplementary Table S5. primer for qualitative RT.PCR

Download Table S5

Supplementary Table S6. primer for qRT-PCR

Download Table S6

Supplementary Table S7. primer for qPCR (ChIP)

Download Table S7

Supplementary Table S8. RNA-FISH probes

Download Table S8

Supplementary Table S9. primers for generation of DNA-FISH probe

Download Table S9
